# Supplementary material for: Epigenetic Patterns in Five-Year-Old Children Exposed to a Low Glycemic Index Dietary Intervention during Pregnancy: Results from the ROLO Kids Study
Source: Nutrients. 2020 Nov 24;12(12):3602. doi: 10.3390/nu12123602 (PMC7760894; doi:10.3390/nu12123602)
Supplement: Supplementary file 1 [file nutrients-12-03602-s001.pdf]

Supplementary Materials

Supplementary Table S1. Principal component analysis of child body composition measurements influencing DNA methylation levels at 5 years of age (n=63)

	**Child Age (years)**	**Weight (kg)**	**Weight Centile**	**Weight SDS**	**Height (cm)**	**Height Centile**	**Height SDS**	**BMI (kg/m²)**	**BMI Centile**	**BMI SDS**	**Chest Circ (cm)**	**Abdomen Circ (cm)**	**Waist: Height Ratio**	**Sum of Skinfolds (n=55)**	**Heart Rate (n=59)**
PC1 correlation	-0.019	0.121	0.137	0.120	0.033	0.035	0.025	0.147	0.170	0.153	0.060	-0.048	-0.030	0.142	-0.243
**PC1 P value**	0.882	0.344	0.285	0.349	0.796	0.785	0.844	0.249	0.182	0.230	0.641	0.711	0.818	0.300	0.064
PC2 correlation	0.034	-0.014	-0.101	-0.048	0.064	0.045	0.060	-0.092	-0.139	-0.110	-0.094	0.054	0.040	-0.099	0.034
**PC2 P value**	0.793	0.912	0.431	0.708	0.618	0.725	0.640	0.471	0.276	0.393	0.463	0.672	0.757	0.471	0.801
PC3 correlation	0.014	-0.075	-0.084	-0.076	-0.147	-0.148	-0.146	0.022	0.052	0.044	-0.105	0.072	0.128	0.131	-0.108
**PC3 P value**	0.916	0.561	0.514	0.554	0.249	0.247	0.255	0.865	0.684	0.735	0.411	0.574	0.318	0.342	0.417
PC4 correlation	-0.194	-0.053	-0.067	-0.051	-0.023	-0.018	0.005	-0.057	-0.090	-0.071	-0.015	-0.067	-0.033	-0.190	0.152
**PC4 P value**	0.128	0.681	0.604	0.692	0.856	0.890	0.971	0.658	0.483	0.578	0.908	0.601	0.798	0.165	0.252
PC5 correlation	-0.076	0.062	0.116	0.083	0.105	0.118	0.120	-0.001	0.043	0.012	0.146	0.044	0.012	0.061	0.011
**PC5 P value**	0.556	0.631	0.366	0.516	0.412	0.358	0.350	0.996	0.740	0.927	0.254	0.729	0.925	0.659	0.932
PC6 correlation	-0.067	0.019	0.014	0.009	-0.072	-0.088	-0.091	0.089	0.131	0.106	0.183	-0.015	0.008	0.062	0.155
**PC6 P value**	0.604	0.882	0.915	0.942	0.574	0.495	0.479	0.490	0.305	0.410	0.151	0.907	0.951	0.651	0.241
PC7 correlation	-0.200	0.040	0.023	0.038	-0.013	0.003	-0.008	0.061	0.092	0.066	0.119	0.070	0.062	-0.075	0.081
**PC7 P value**	0.116	0.754	0.860	0.765	0.920	0.982	0.948	0.634	0.473	0.605	0.352	0.587	0.628	0.587	0.542
PC8 correlation	-0.049	-0.105	-0.086	-0.092	-0.229	-0.201	-0.205	0.065	0.056	0.057	-0.163	-0.070	0.021	0.041	-0.067
**PC8 P value**	0.702	0.412	0.501	0.473	0.070	0.115	0.108	0.611	0.661	0.655	0.203	0.588	0.869	0.769	0.616
PC9 correlation	0.111	-0.016	-0.007	-0.019	0.125	0.129	0.106	-0.141	-0.095	-0.110	-0.153	-0.105	-0.151	0.104	0.081
**PC9 P value**	0.388	0.902	0.955	0.885	0.329	0.314	0.407	0.272	0.459	0.392	0.231	0.412	0.237	0.452	0.541
PC10 correlation	0.039	0.106	0.096	0.098	0.154	0.115	0.127	0.015	-0.012	0.019	0.025	-0.066	-0.119	-0.011	-0.041
**PC10 P value**	0.764	0.407	0.456	0.446	0.229	0.369	0.320	0.904	0.927	0.885	0.843	0.610	0.351	0.937	0.756

BMI: Body Mass Index (kg/m²), Circ: circumference, PC: Principal Component, SDS: Standard deviation score, *Significant at $P < 0.05$
